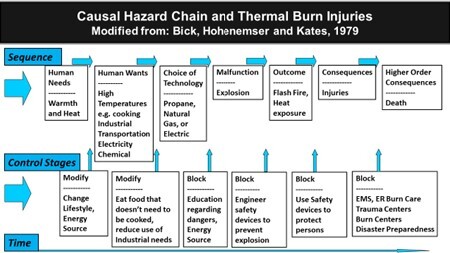# 745 Old Dog, New Tricks? Applying the Causal Hazard Chain Model to Burn Prevention and Injuries

**DOI:** 10.1093/jbcr/irae036.288

**Published:** 2024-04-17

**Authors:** Randy D Kearns, Jeffrey E Carter, Ernest Grant, William L Hickerson

**Affiliations:** University of New Orleans, New Orleans, LA; University Medical Center (LSU Health), New Orleans, LA; Duke University School of Nursing, Chapel Hill, NC; University of Tennessee (Retired), Memphis, TN; University of New Orleans, New Orleans, LA; University Medical Center (LSU Health), New Orleans, LA; Duke University School of Nursing, Chapel Hill, NC; University of Tennessee (Retired), Memphis, TN; University of New Orleans, New Orleans, LA; University Medical Center (LSU Health), New Orleans, LA; Duke University School of Nursing, Chapel Hill, NC; University of Tennessee (Retired), Memphis, TN; University of New Orleans, New Orleans, LA; University Medical Center (LSU Health), New Orleans, LA; Duke University School of Nursing, Chapel Hill, NC; University of Tennessee (Retired), Memphis, TN

## Abstract

**Introduction:**

From burn injuries to widespread catastrophe and calamity, fire has been associated with unexpected death and destruction. Worldwide, 8,378,122 were hospitalized in 2019 with a burn injury.1 Approximately 180,000 die as a result of a burn injury annually.2 There is a known or predictable sequence of events that precede injury and death with most burn injuries. Understanding the risks, causality, and nature of burn injury prevention is essential to prevent catastrophic events in the workplace,3,4 following a natural disaster,5 or in the home.

**Methods:**

For this work, we applied the Causal Hazard Sequence (CHS)6-9 relying on the standard CHS series of events:

1. Human Needs

2. Human Wants

3. Choice of Technology

4. Malfunction

5. Outcomes

6. Consequences

7. Higher Order Consequences

For each of the seven steps in the series of events, the "Control Stage" of the CHS model requires that you consider "modification" of "human wants" and "human needs." As the series moves into the "choice of technology" phase, with growing complexity, the option is to either "change" the technology chosen or create methods to "block" or reduce the impact of unintended outcomes that could arise. The block takes on increasingly complex solutions as the risk from the chosen technology also becomes increasingly complex.

**Results:**

A predictable sequence of events that precede morbidity and mortality associated with burn injuries can be derived by analyzing the root cause of a burn injury. While the timeline may vary, the progression can be plotted somewhat linearly. This graphical model is offered to understand better the risks and hazards associated with fire and heat sources related to burn prevention and burn injury. Furthermore, the model includes the response to and the care of a burn patient and the checks and balances that should be in place to avoid, prevent or minimize injury and deaths.

Understanding the cause of a burn injury starts with the "needs, wants, and technology" that was involved in a burn injury. By examining what conditions amplify these events or could be a point of intervention, the CHS process can aid with mapping out a strategy to alter or break the chain of events and thereby reduce risk. While not all interventions we have discussed are suitable, the model offers a more thorough review of the threat of a burn injury compared to the human needs associated with fire and heat.

**Conclusions:**

The model used with the CHS provides users and the audience with a simple, graphical representation to understand, more quickly and clearly, the risks and the opportunities to reduce those risks when dealing with heat sources.

**Applicability of Research to Practice:**

The causal hazard chain and its rather simple yet deliberate manner to describe how our quest to use fire/heat has been both of great benefit and where there are opportunities to prevent injury. The aim of this adaptation of the causal hazard chain in the field of burn care and burn prevention is another way we can explain burn prevention strategies.